# Idiopathic granulomatous mastitis: A retrospective cohort study between 44 patients with different treatment modalities

**DOI:** 10.1016/j.amsu.2018.11.001

**Published:** 2018-11-09

**Authors:** Prakasit Chirappapha, Panya Thaweepworadej, Chairat Supsamutchai, Namsiri Biadul, Panuwat Lertsithichai

**Affiliations:** a Department of Surgery, Faculty of Medicine, Ramathibodi Hospital, Mahidol University, Bangkok, Thailand; b Department of Surgery, Bangkok Metropolitan Administration General Hospital, Bangkok, Thailand

**Keywords:** Idiopathic granulomatous mastitis, IGM, Mastitis, Granulomatous lobular mastitis

## Abstract

**Background:**

Idiopathic granulomatous mastitis (IGM) is an uncommon benign chronic inflammatory disease which can clinically and radiographically mimic abscess or breast cancer. Definitive diagnosis was made by histopathology and exclusion of an identifying etiology. Optimal treatment has not been yet established. The aim of this study was to report and describe the clinical signs, radiological findings, managements, clinical course, and clinical outcomes after treatment of IGM.

**Method:**

We retrospectively studied IGM medical records of 44 patients in our institute collected from March 1990 to October 2016. The patient characteristics, clinical presentations, radiological findings, microbiological workups, tissue pathology, treatment modalities, outcomes, and follow-up data were reviewed and analyzed. The success rate, recurrence rate and time-to-healing were compared focusing on the treatment modalities to find the proper treatments for IGM patient.

**Results:**

Forty-four patients were diagnosed as IGM. The median follow-up time was 20.73 months ranging from 1.26 to 118.8 months while the median time of the diagnosis was 21 days ranging from 2 to 246 days. Due to the follow-up period, only thirty-nine patient data were used for the analysis. In the first setting, 30 patients were treated by surgery, 6 patients were treated by using steroid while other 3 patients were treated by other different treatments. Only 25 from 39 patients (64.10%) were cured by the first modality. The overall median time-to-healing was 84 days while the medians of time-to-healing treated by surgery, steroid and the rest were 75, 114.5, and 238 days respectively. The surgical treatment had the shortest time-to-healing but not statistically significant (p = 0.23). Thirteen patients out of twenty-five (52%) had wound complications after performing an excision. Lastly, five patients out of thirty-nine (12.82%) had recurrence.

**Conclusion:**

IGM is an uncommon benign disease which is hardly distinguished from malignancy. There is not a significant difference among treatment modalities in term of time-to-healing and recurrence of disease. The result shows that surgery is outperformed by the shortest healing time. However, the surgical treatment must be chosen with careful due to high rate of wound complications. Multimodality treatment is recommended as the proper treatments for IGM patient.

## Highlights

• No difference between each treatment modalities for IGM in term of time-to-healing and recurrence of disease.
• The success rate by wide local excision alone was 65.22% and had the shortest healing time.
• But wound complication rate from surgery was quite high (52%) even in the case performing mastectomy.
• Multimodality treatment is required in one-third of the patients.

## 1 Background

IGM is an uncommon benign chronic inflammatory disease of unknown etiology which can clinically and radiographically mimic abscess or breast cancer. IGM was first described among benign breast diseases in 1972 by Kessler and Wolloch [1]. Many case series have been reported since then. Variety of IGM incidences have been reported while the true is still unknown. IGM large series are mostly found in Asia and Mediterranean region [2]. This may indicate that the higher prevalence of disease is related to an ethnicity. Baslaim et al. reported 20 IGM cases from 1241 cases of benign breast disease specimen from tertiary center in Saudi Arabia over a period of 10 years [3]. The IGM largest series of more than 700 cases was recently reported from Turkey [4]. Ocal et al. study showed that IGM typically affects women of childbearing age, who have a recent history of breast feeding records [5].

By clinically, most patient were presented with palpable mass, local inflammation, and fistula formation which can't be distinguished from breast cancer [6,7]. Clinical and radiological findings of IGM are varied and non-specific. To diagnosis, a histopathological examination is mandatory. Generally, a definitive diagnosis is made by histopathological presence of non-caseating granulomas confined within the breast lobules and exclusion of any known etiological factors such as infections, duct ectasis, and autoimmune process [8,9]. Pathogenesis of IGM are comprised of multiple non-specific lobulitis which causes lymphoplasmocytic infiltration, and granulomatous formation. Many factors have been concerned including hormonal imbalance, autoimmunity, unknown microbiological agents, smoking, and antitrypsin deficiency.

Although IGM is a benign disease, high rate of recurrence was variously reported between 5% and 50% [4]. Many studies have tried to evaluate etiological factors related with recurrence outcome but the results were unsatisfied [[2], [3], [4]]. Due to an unknown etiology and rareness, diagnosis and treatment is still a challenge. Optimal treatment has not been yet established, while medical therapy, wide local excision, abscess drainage, and expectant management are currently the favored treatment options. Some of the medical treatments have been reported including antibiotics, systemic steroids, non-steroidal anti-inflammatory drugs (NSAIDs), and immunosuppressive agents, such as methotrexate (MTX) and azathioprine [[10], [11], [12], [13]]. Delayed wound healing and recurrence of disease have been reported after surgical excision. Many studies reported comparable outcome between surgery and steroid therapy with less scar and invasiveness suggested that treatment with steroid might be the first choice for IGM [[14], [15], [16], [17]].

Th clinical signs, radiological findings, managements, clinical course, and clinical outcomes after treatment of IGM will be reported and described in this study by analyzing the patient characteristics, clinical presentations, radiological findings, microbiological workups, tissue pathology, treatment modalities, outcomes, and follow-up data.

## 2 Patients and methods

We retrospectively reviewed medical records in our institute collected from March 1990 to October 2016. During that period, forty-four female and one male were diagnosed as granulomatous mastitis with pathological proven. One patient was excluded from the study due to positive AFB staining and data from 5 patients were excluded from a comparative analysis due to very short follow-up period. Patient characteristics, clinical presentations, radiological findings, microbiological workups, tissue pathology, treatment modalities, outcomes, and follow-up data were reviewed. All patients were performed tissue diagnosis before treatment. The pathological criteria for diagnosis of IGM were presence of the histopathological features of non-caseous granulomatous inflammation on breast lobule and absence of an identifying etiology. All specimens were sent for microbiological analysis with staining and cultures for bacteria, fungus, and mycobacteria. Treatment approaches, such as antibiotics and steroid with or without surgery were reviewed. All patients with disease-free follow-up period were included in the study. All patients were classified into 3 groups based on the first treatment setting: performing surgery, using steroid and applying other different treatments. Main outcomes in our study were curative of disease and time-to-healing. Curative of disease was defined as complete healing of all surgical wound and no inflammatory process was observed. Recurrence of disease was defined as a recurrence of symptoms such as pain, mastitis, or abscess more than 3 months after the disease is cured. All procedures were performed by surgeons from our Breast and Endocrinology unit. This study was approved by the institutional review board. The protocol had been registered at Thai Clinical Trials Registry (TCTR) with the identification number TCTR20180828005.

Descriptive statistics were used to describe demographic data, choice of treatment, complications and treatment outcomes. All quantitative data were described in the table as percentage and median ± interquartile range (IQR). Differences outcomes among three treatments modalities were analyzed concerning to co-morbidities and patient characteristics. Wilcoxon-Mann-Whitney test and Kruskal-Wallis one-way analysis of variance was used to test the differences of time-to-healing and the Fisher exact probability test was used to test an association between patient characteristics and treatment outcomes All statistical analysis was performed by statistician with STATA version 14.0.

## 3 Results

The characteristics of 44 patients showed that the average of patient age was 38 years old ranging from 21 to 81. Most patients (66.7%) were between 21 and 40 years old. About one-third of patients were obese (16 patients, 36.36%). Thirty patients had history of pregnancy but only one of them had a recent breast-feeding history record. Thirty-one percent of our patients were associated with an oral contraceptive pill. Only 8 patients (18.18%) had co-morbidities, and 3 of them had history of tuberculosis. Baseline patient characteristics were shown in Table 1.

**Table 1:** Baseline characteristics from 44 patients diagnosed with Idiopathic Granulomatous Mastitis (IGM).

	Surgery (N = 33)	Steroid (N = 6)	Others (N = 5)	Total
N (%) or Mean±SD				
Age (year)				
<20	0	0	0	0
20-40	21 (63.64)	3 (50)	5 (100)	29 (65.91)
41-60	9 (27.27)	3 (50)	0	12 (27.27)
61-80	2 (6.06)	0	0	2 (4.55)
>80	1 (3.03)	0	0	1 (2.27)
BMI (kg/m^2^)	25.0 ± 5.31	23.84 ± 3.68	24.91 ± 3.79	25.18 ± 4.84
<20	4 (12.12)	0	1 (0.2)	5
20-25	8 (24.24)	4 (66.67)	1 (0.2)	13
25-35	11 (33.33)	2 (33.33)	2 (0.4)	15
>35	1 (3.03)	0	0	1
Missing	9	0	1	10
Prior pregnancy	22 (66.67)	4 (66.67)	4 (80)	30 (68.18)
Recent lactation	1 (3.03)	0	0	1 (2.27)
Contraceptive pills use	11 (33.33)	4 (66.67)	0	14 (31.82)
Smoking	1 (3.03)	0	0	1 (2.27)
Co-morbidities	7 (21.21)	1 (16.67)	0	8 (18.18)
Diabetes Mellitus (DM)	2 (6.06)	0	0	2 (4.55)
Hypertension (HT)	2 (6.06)	1 (16.67)	0	3 (6.82)
Immune-related disease	0	0	0	0
History of tuberculosis	3 (9.1)	0	0	3 (6.82)
Others	4 (12.12)	0	0	4 (9.09)

In this study, all patients presented with unilateral lesion. Most of the patient symptoms (more than 90%) were palpable mass and abscess located on an upper part of breast. Radiological findings were usually non-specific and mostly suspicious as malignancy. Twenty patients from twenty-seven were reported as BIRADS 4 and 5 category. The median time to diagnosis was 21 days ranging from 2 to 246 days. The data were presented in Table 2.

**Table 2:** Clinical presentation from 44 patients diagnosed with IGM.

	N (%)
Signs and symptoms	
Mass	33 (75)
Ulcer	1 (2.27)
Mastitis/Abscess	11 (24.44)
Fistula	2 (4.55)
Pain	1 (2.27)
Number of mass lesions	
0	2 (4.55)
1	37 (84.09)
2	2 (4.55)
Side (right: left)	22: 22
Location of lesions	
Retroareolar	4 (9.09)
Upper-inner quadrant	14 (31.82)
Upper-outer quadrant	15 (34.09)
Lower-inner quadrant	6 (13.64)
Lower-outer quadrant	5 (11.36)
BIRADS	
2	3 (6.81)
3	4 (9.09)
4	18 (40.91)
5	2 (4.55)
Miss data	17 (38.64)

**Fig. 1:**
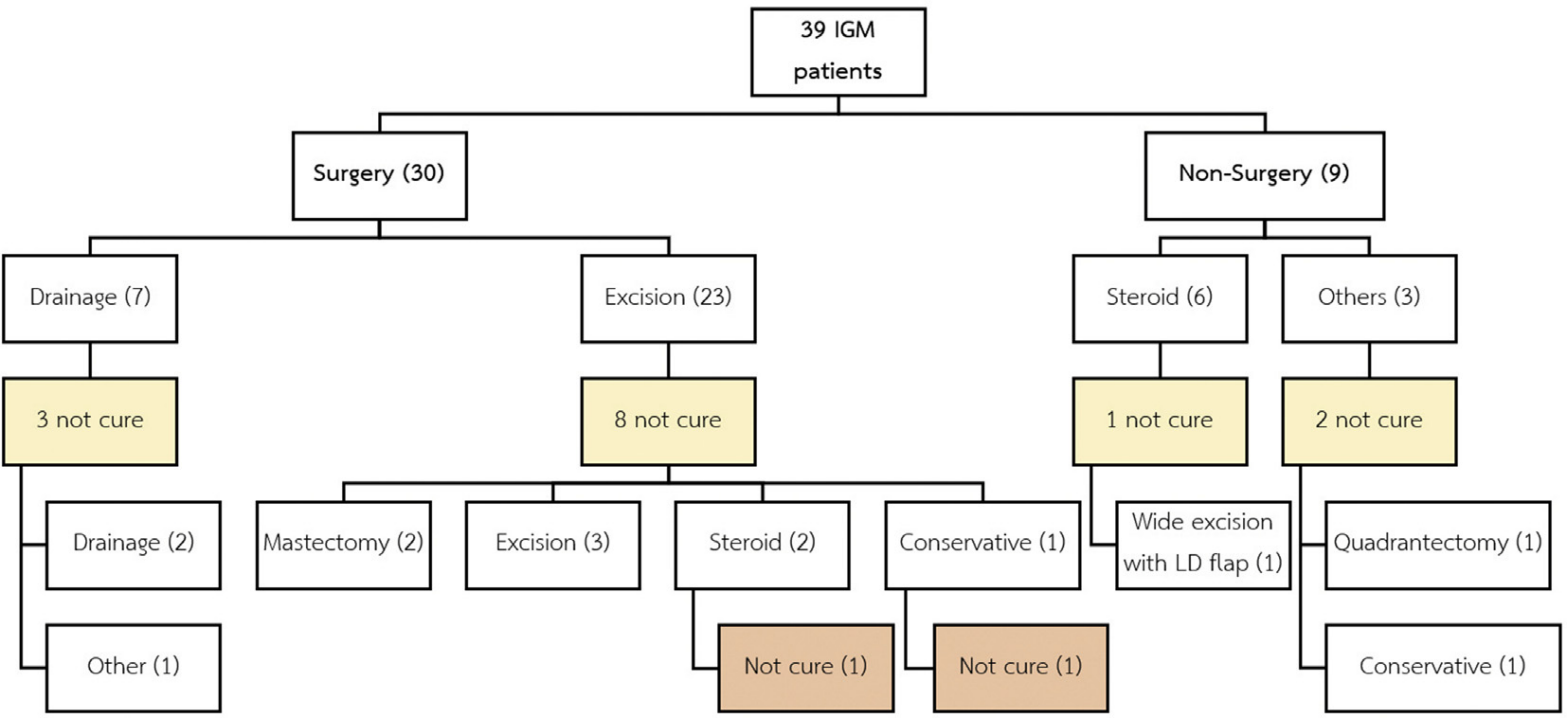
Treatment modalities performed in 39 patients with IGM.

In comparative analysis, thirty-nine patients were categorized by the first treatment setting and analyzed by an intention-to-treat basis. The median of the follow-up time was 20.73 months ranging from 1.26 to 118.8 months. Most of the patients (76.9%) were treated by surgical intervention in the first setting (30 patients were treated by surgery, and 6 patients were treated by using systemic steroid while other 3 patients were treated by other different treatments). Only 25 from 39 patients (64.10%) were cured by the first modality. Wide local excision was performed as the first treatment setting in major of the patients (59%). The success rate by the first excision alone was 65.22%. Nine patients were treated by using corticosteroid and overall success rate was 77.78%. Three patients out of thirty-nine were classified as “others” treatment group. All of them had history of pulmonary tuberculosis and developed mastitis with granulomatous inflammation. All microbiological tests were negative. They were failed after treatment with antituberculosis by another department. Two patients were not cured after treatments. They developed a wound disruption and delay wound healing. One of them was treated by using a 60 mg of oral Prednisolone per day. The wound was healed but the patient still had some inflammation and then she was lost to follow-up after 7 months of treatments. The rest of them was treated by conservative treatment, but the wound was not healed, and she lost to follow-up after 9 months of treatments. Summary of the treatment modalities were shown in Fig. 1.

The overall median of time-to-healing was 84 days while the median of time-to-healing treated by surgery, steroid and the rest were 75, 114.5 and 238 days respectively. Surgical treatment had the shortest time-to-healing compared with corticosteroid and the rest of treatments (the median of 75, 114.5 and 238 days respectively, but the result was not statistically significant (p = 0.23) (Table 3). Thirteen patients out of twenty-five (52%) that were performed by local excision including one patient that was performed by mastectomy had wound complications such as surgical site infection, wound disruption, and delay wound healing. There was no non-surgical morbidity and mortality in our series. Five patients out of thirty-nine (12.82%) had recurrence of IGM. Four patients were treated by surgery and the rest was treated by steroid in the first setting. All of them were treated with non-surgical approach. Three patients were self-limited, and the rest of them were successfully treated with corticosteroid. The median of time to recurrence was 280 days ranging from 164 to 358 days in our series. There was no association between patient characteristics such as, age group, BMI, co-morbidities and outcomes of the treatments in our series. But elderly patients and the patient with co-morbidity tended to take longer time to heal in the study (Table 4).

**Table 3:** Time-to-healing between treatment modalities in 39 patients with IGM.

Treatment	Time-to-healing (day)						
	n	median	iqr	Percentile 25	Percentile 75	(Min, max)	P-value
Surgery	29^a^	75	115	35	150	10, 416	0.2274
Steroid	6	114.5	105	42	147	42, 416	
Others	3	238	236	121	357	121, 357	

a Missing data in 1 patient.

**Table 4:** Association between patient characteristics and treatment outcome in 39 patients with IGM.

Time-to-healing (day)					
		n	Median	iqr	P - value
Age (year)	<20	1	416	0	0.071
	21–40	24	60.5	86.5	
	41–60	10	132.5	151	
	61–80	2	391	68	
	>80	1	145	0	
Prior pregnancy	No	10	49.5	104	0.427
	Yes	26	113.5	154	
Contraceptive pills use	No	24	87.5	141.5	0.736
	Yes	11	130	196	
BMI (kg/m^2^)	<18.5	1	91	0	0.524
	18.5–25.0	15	64	98	
	25.01–30.0	8	168	157.5	
	>30.0	6	32	37	
Co-morbidities	No	30	76	105	0.075
	Yes	7	204	244	
DM	No	35	91	162	0.470
	Yes	2	77.5	135	
HT	No	35	91	162	0.420
	Yes	2	77.5	135	
Old tuberculosis	No	34	80.5	105	0.012*
	Yes	3	369	57	

## 4 Discussion

IGM is a rare benign breast disease. We have found only 44 patients over the past 15 years. Most of them were diagnosed within the past 5 years reflecting underdiagnoses in the early of our study. Although it was first described more than four decades, an etiology and pathophysiology of IGM is still unknown. Some literature described an association with contraceptive pills, lactation, hyperprolactinemia, smoking and Corynebacterium infection [2,[18], [19], [20], [21]]. Higher severity and longer duration of disease in puerperal period was shown in one study [22]. In our series, more than 90% of patients were in child-bearing age and only 1 patient presented during breast feeding. We have found no relationship with other factors such as smoking, or Corynebacterium infection in our study.

The most important issues in diagnosis is to distinguish IGM from cancer. Seventy-five percent of the patients presenting with palpable mass in our series, and nearly half of them were suspicious and highly suspicious for malignancy by imaging made histopathologically diagnosis might play the major role in diagnosis. The diagnosis of IGM is made by an exclusion, we suggest that staining and cultures for bacteria, fungus, and mycobacteria should be done in all patients especially in high prevalence area of tuberculosis.

**Fig. 2:**
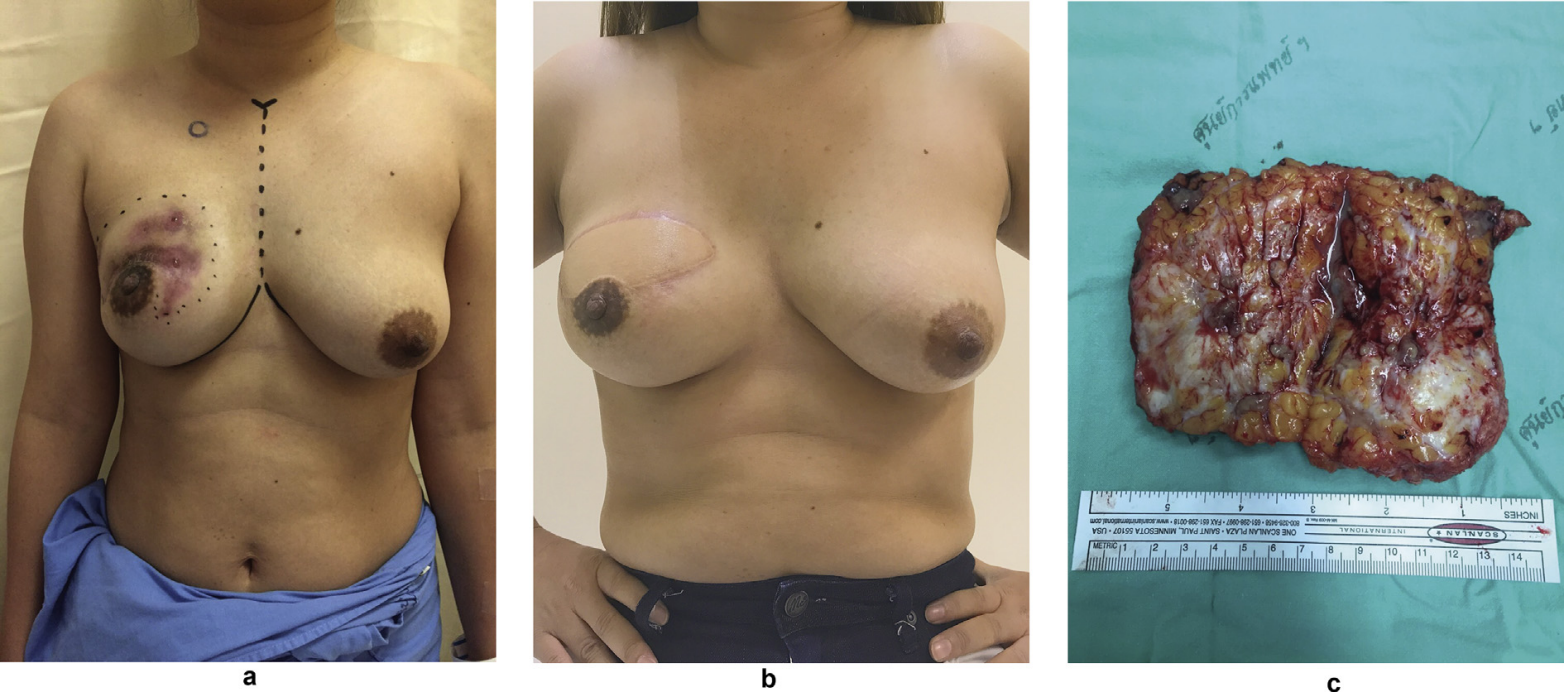
Pictures of the patient with IGM performing immediate Latissimus dorsi (LD) flap reconstruction., a. Pre-operative view, granulomatous infection involved in upper half of right breast. b. Post-operative view, 6 months after immediate LD flap reconstruction at right breast. c. Gross specimen after totally removed from right breast.

The treatment of IGM is still challenging as there is no standard of treatment nowadays. Uysal et al. was reported multicenter retrospective study including 720 IGM patients from 22 centers in Turkey [4]. In their series, more than 50% of patients were treated with multimodalities approach followed by using corticosteroid in 39%, and surgery alone in 8% of patients. Overall recurrence was 17% from their series. Wide local excision with or without corticosteroid therapy is the mainstay of treatment in many literature [9,[23], [24], [25]]. Local excision has shown the shortest healing time, but delay of wound healing and high recurrence rate were reported vary in 10–50% and 8–38%, respectively. Our study also shows the shortest healing time after surgery. But surgical treatment had 52% of wound complications and 13.3% recurrence rate. Even high rate of surgical complications, some authors reported successful performing tissue reconstruction after surgery in IGM [9,26,27]. We performed an autologous tissue reconstruction in one patient after 3 months treatment with corticosteroid. Her lesion was partially response with less inflammation and pain, but she still suffered from wound fistula and discharge. Her lesion was very large and involved in 2 quadrants of right breast. We discussed choices of the treatment with the patient and our multidisciplinary care team. The patient decided to performed surgery with immediate reconstruction. Due to high rate of wound complications, we preferred autologous tissue more than implant-based reconstruction to prevent the surgical site infection and implant exposure. The operation was success without post-operative complication. Surgical wound was completely healed in 2 weeks without relapse or recurrence of disease. Post-operative result was shown in Fig. 2. However, we suggest a careful selection of patient and breast reconstruction should be done in highly selected group of patients. The treatment options should be discussed with the patient and medical team.

Yukawa et al. reported a series of 13 patients treated without corticosteroid [28]. In their series, 11 of 13 patients required limited drainage of abscess and time to resolution was 4–28 months. In our series, none of the patients was conservative in the first setting. Five patients out of fifteen that were failed or recurred from previous treatment were successfully treated by conservative approach. It was confirmed that the natural history of IGM might be self-limiting.

Due to rareness of disease, there is no prospective study comparing each modalities of treatment. Even the high rate of wound complications, surgical treatment has a shorter time of treatment and might has some role in the treatment of IGM in highly selected patient. One-third of the patients need multimodality treatment. Further study is required to answer the question that which options is the best.

## Ethical approval

Committee on Human Rights Related to Research Involving Human Subjects Faculty of Medicine Ramathibodi Hospital, Mahidol University Protocol Number: ID 08-59-52.

## Sources of funding

No grants or financial support were received by any of the authors in relation to this study or to the writing of this article.

## Author contribution

1. Asst. Prof. Prakasit Chirappapha

Department of Surgery, Faculty of Medicine Ramathibodi Hospital, Mahidol University, Bangkok, Thailand

Writing manuscript and interpretation of data

2. Dr. Panya Thaweepworadej

Department of Surgery, Faculty of Medicine Ramathibodi Hospital, Mahidol University, Bangkok, Thailand

Interpretation of data

3. Dr. Chairat Supsamutchai

Department of Surgery, Faculty of Medicine Ramathibodi Hospital, Mahidol University, Bangkok, Thailand

Correspondent, interpretation of data and analysis

4. Dr. Namsiri Biadul

Department of Surgery, Faculty of Medicine Ramathibodi Hospital, Mahidol University, Bangkok, Thailand

Acquisition of data

5. Assoc. Prof. Panuwat Lertsithichai

Department of Surgery, Faculty of Medicine Ramathibodi Hospital, Mahidol University, Bangkok, Thailand

Acquisition of data

## Conflicts of interest

All authors have no any financial and personal relationships with other people or organization that could inappropriately influence (bias) their work.

## Research registry number

The protocol had been registered at Thai Clinical Trials Registry (TCTR). The identification number is TCTR20180828005.

## Guarantor

Dr. Chairat Supsamutchai (Correspondent author), Department of Surgery, Faculty of Medicine, Ramathibodi Hospital, Mahidol University, Bangkok, Thailand, pogeneral2007@hotmail.com.

## Consent

Written informed consents were obtained in all patients for publication of this case report and accompanying images. A copy of the written consent is available for review by the Editor-in-Chief of this journal on request.

## Provenance and peer review

Not commissioned, externally peer reviewed.

## Appendix A Supplementary data

STROCSS_statement_checklist.pdf STROCSS_statement_checklist.pdf
